# ChatGPT's Ability to Assess Quality and Readability of Online Medical Information: Evidence From a Cross-Sectional Study

**DOI:** 10.7759/cureus.42214

**Published:** 2023-07-20

**Authors:** Roei Golan, Sarah J Ripps, Raghuram Reddy, Justin Loloi, Ari P Bernstein, Zachary M Connelly, Noa S Golan, Ranjith Ramasamy

**Affiliations:** 1 Department of Clinical Sciences, Florida State University College of Medicine, Tallahassee, USA; 2 Herbert Wertheim College of Medicine, Florida International University, Miami, USA; 3 Department of Urology, Montefiore Medical Center, Bronx, USA; 4 Department of Urology, New York University Langone Health, New York, USA; 5 Department of Surgery, Louisiana State University Health Shreveport, Shreveport, USA; 6 Department of Psychology, University of Florida, Gainesville, USA; 7 Department of Urology, Desai Sethi Urology Institute, Miami, USA

**Keywords:** healthcare ai and robotics, shock wave therapy, online medical information, readability, chatgpt, artificial intelligence in medicine

## Abstract

Introduction

Artificial Intelligence (AI) platforms have gained widespread attention for their distinct ability to generate automated responses to various prompts. However, its role in assessing the quality and readability of a provided text remains unclear. Thus, the purpose of this study is to evaluate the proficiency of the conversational generative pre-trained transformer (ChatGPT) in utilizing the DISCERN tool to evaluate the quality of online content regarding shock wave therapy for erectile dysfunction.

Methods

Websites were generated using a Google search of “shock wave therapy for erectile dysfunction” with location filters disabled. Readability was analyzed using Readable software (Readable.com, Horsham, United Kingdom). Quality was assessed independently by three reviewers using the DISCERN tool. The same plain text files collected were inputted into ChatGPT to determine whether they produced comparable metrics for readability and quality.

Results

The study results revealed a notable disparity between ChatGPT's readability assessment and that obtained from a reliable tool, Readable.com (p<0.05). This indicates a lack of alignment between ChatGPT's algorithm and that of established tools, such as Readable.com. Similarly, the DISCERN score generated by ChatGPT differed significantly from the scores generated manually by human evaluators (p<0.05), suggesting that ChatGPT may not be capable of accurately identifying poor-quality information sources regarding shock wave therapy as a treatment for erectile dysfunction.

Conclusion

ChatGPT’s evaluation of the quality and readability of online text regarding shockwave therapy for erectile dysfunction differs from that of human raters and trusted tools. Therefore, ChatGPT's current capabilities were not sufficient for reliably assessing the quality and readability of textual content. Further research is needed to elucidate the role of AI in the objective evaluation of online medical content in other fields. Continued development in AI and incorporation of tools such as DISCERN into AI software may enhance the way patients navigate the web in search of high-quality medical content in the future.

## Introduction

As technology advances at a rapid pace, the importance of health literacy is increasing, as patients must comprehend complex medical information to make informed decisions about their health. Inadequate health literacy is strongly associated with social determinants of health, such as employment status, lifetime income, and education [1,2]. The readability of medical content, defined as the simplicity by which written materials can be understood, is paramount to breaking barriers among all patient populations. Poor readability of medical content can have detrimental effects, such as patient misunderstanding, and may subsequently impact treatment decisions. Recent literature suggests that online information and social media content regarding men’s health is of poor quality [3,4]. Our group previously assessed the readability and quality of online content regarding shockwave therapy for erectile dysfunction using Readable software (Readable.com, Horsham, United Kingdom) and DISCERN instrument, respectively [3,5,6]. We found that content generated from private medical practices was as readable as articles from universities or news media, but of lower scientific quality as evidenced by lower DISCERN scores [3].

Artificial intelligence (AI) is the capability of computer systems to carry out tasks that conventionally require human intelligence, such as visual perception and decision-making. OpenAI, an AI research laboratory, developed the large language model conversational generative pre-trained transformer (ChatGPT) for public use, which has been trained on tremendous amounts of text data and employs deep learning algorithms. ChatGPT imitates and interprets human language with high precision and has been demonstrated to perform well for a variety of broad purposes, including performance on standardized examinations, such as the USMLE Step 1, research queries, drug development, and discovery [7-13]. 

The integration and incorporation of AI and web search capabilities are rapidly being established. Free AI web-assistant tools such as Monica and HARPA AI have been developed to streamline the process of web browsing. These tools integrate ChatGPT into the web browsing sidebar, providing users with summaries of webpages in a simplified format after conducting an initial Google search. Although these tools enhance efficiency and enable users to access a larger volume of information in less time, the ability of ChatGPT to evaluate the readability and quality of medical text and detect biases remains uncertain. Evaluating the ability of ChatGPT to determine the quality of online medical content is currently imperative and will become increasingly important, as patients increasingly rely on the internet and social media for medical information.

Both Readable software and the DISCERN tool are established and trusted standards in health communication [3,5,6]. Widely used and validated by experts, they offer a reliable framework for evaluating medical text readability and quality [3,5,6]. To our knowledge, no previous research has been conducted evaluating ChatGPT’s proficiency in utilizing Readable software and the DISCERN tool to appraise medical text readability and quality. Thus, the aim of this study was to feed online medical text previously collected from our group’s prior study into ChatGPT and examine whether it produced comparable outcomes for both readability and quality. We hypothesized that ChatGPT's findings would mirror those of our prior investigation given its high performance across a multitude of tasks in various sectors.

## Materials and methods

In our previous study, we performed a Google search of “shock wave therapy for erectile dysfunction” with location filters disabled [3]. All websites containing articles on the first page of Google were copied and downloaded as plain text files. Of the 10 websites on the first page of the Google search, one website was excluded since it led to a scientific article. Readability was evaluated using Readable software (Readable.com, Horsham, United Kingdom). Quality of content was scored independently by three authors using the DISCERN questionnaire tool, which investigates the quality of information regarding treatment options for diseases or conditions.

The same plain text files collected in our previous study were used in the current study but were instead entered into ChatGPT. ChatGPT Mar23 version was used on May 2, 2023. Quality was assessed by instructing ChatGPT to answer the 16 questions from the DISCERN tool (which had been done manually by three authors in our previous study [3]). We also instructed ChatGPT to assess the readability of the entered text. The prompt was inserted three times into new chat boxes each time.

Instructions for readability assessment

The following was inputted into ChatGPT to assess the readability of texts:

"Using the article copied, rate the readability of the article using the following scales: Flesch-Kincaid level, Gunningfox index, Coleman-Liau index, SMOG index, Automated readability index, FORCAST grade level, Flesch reading ease."

Instructions for quality assessment

The following was inputted into ChatGPT to assess the quality of texts:

"Using the article copied, answer the 16 questions below. Your answers should only be a number on a scale of 1-5. 1. Are the aims clear? 2. Does it achieve its aims? 3. Is it relevant? 4. Is it clear what sources of information were used to compile the publication (other than the author or producer)? 5. Is it clear when the information used or reported in the publication was produced? 6. Is it balanced and unbiased? 7. Does it provide details of additional sources of support and information? 8. Does it refer to areas of uncertainty? 9. Does it describe how each treatment works? 10. Does it describe the benefits of each treatment? 11. Does it describe the risks of each treatment? 12. Does it describe what would happen if no treatment is used? 13. Does it describe how the treatment choices affect the overall quality of life? 14. Is it clear that there may be more than one possible treatment choice? 15. Does it provide support for shared decision-making? 16. Based on the answers to all of the above questions, rate the overall quality of the publication as a source of information about treatment choices."

Statistics

The collected DISCERN scores from the three independent reviewers were assessed to ensure that no significant differences existed between raters, and the scores were then averaged.

The ChatGPT-calculated DISCERN scores were compared to our previously determined findings assessed by three humans using a student t-test. In addition, the ChatGPT-calculated readability scores were compared to our previously determined findings assessed by Readable.com.

A one-way ANOVA with a Tukey post-hoc analysis was utilized to determine whether the ChatGPT-calculated DISCERN scores were statistically different between articles from academic versus private sources.

## Results

Overall, ChatGPT was able to successfully evaluate the quality of the nine texts using the 16-question DISCERN tool. Additionally, ChatGPT was able to successfully evaluate the readability of the nine texts using seven different readability scales. While ChatGPT often generated different DISCERN score values for the same article/question upon repeat query, there were notably no significant differences between the DISCERN scores among each of the three queries performed by ChatGPT, indicating strong inter-rater reliability and concordance (p=0.94), suggesting that ChatGPT's answers are precise.

Six out of 16 (37.5%) DISCERN questions (questions 5, 9, 10, 11, 12, and 15) yielded scores that were statistically different between AI and human reviewers (p<0.05). For these six questions, ChatGPT suggested that the quality of the text was significantly higher than the score of the three human reviewers in five out of the six (83.3%) DISCERN questions (p<0.05) (Table 1). This suggests ChatGPT's performance in assessing the quality of textual content was inadequate, and it did not demonstrate consistency with the standards set by human evaluators.

**Table 1:** Student's t-test was used to compare the mean DISCERN ratings between three human reviewers and ChatGPT. Six out of 16 of the DISCERN questions were rated differently when comparing three human reviewers to ChatGPT. * indicates p-values less than 0.05

TABLE 1 DISCERN Questions	Reviewer	N	Mean	Std. Error Mean	p-value
1. Are the aims clear?	Human	27	4.222	0.180	0.283
	ChatGPT	27	4.444	0.097	
2. Does it achieve its aims?	Human	27	4.074	0.168	0.077
	ChatGPT	27	3.704	0.117	
3. Is it relevant?	Human	27	4.259	0.147	0.183
	ChatGPT	27	4.519	0.124	
4. Is it clear what sources of information were used to compile the publication (other than the author or producer)?	Human	27	3.037	0.322	0.597
	ChatGPT	27	2.852	0.127	
5. Is it clear when the information used or reported in the publication was produced?	Human	27	2.741	0.327	0.032*
	ChatGPT	27	3.519	0.112	
6. Is it balanced and unbiased?	Human	27	2.963	0.253	0.27
	ChatGPT	27	3.296	0.158	
7. Does it provide details of additional sources of support and information?	Human	27	2.852	0.281	0.491
	ChatGPT	27	2.630	0.152	
8. Does it refer to areas of uncertainty?	Human	27	2.963	0.299	0.353
	ChatGPT	27	3.296	0.191	
9. Does it describe how each treatment works?	Human	27	3.667	0.207	0.002*
	ChatGPT	27	4.407	0.096	
10. Does it describe the benefits of each treatment?	Human	27	3.593	0.202	0.016*
	ChatGPT	27	4.148	0.088	
11. Does it describe the risks of each treatment?	Human	27	2.333	0.316	0.001*
	ChatGPT	27	3.667	0.151	
12. Does it describe what would happen if no treatment is used?	Human	27	2.407	0.187	0.005*
	ChatGPT	27	3.185	0.185	
13. Does it describe how the treatment choices affect the overall quality of life?	Human	27	3.333	0.151	0.766
	ChatGPT	27	3.259	0.197	
14. Is it clear that there may be more than one possible treatment choice?	Human	27	3.296	0.244	0.309
	ChatGPT	27	3.630	0.214	
15. Does it provide support for shared decision-making?	Human	27	3.593	0.222	0.01*
	ChatGPT	27	2.852	0.166	
16. Based on the answers to all of the above questions, rate the overall quality of the publication as a source of information about treatment choices	Human	27	3.148	0.271	0.094
	ChatGPT	27	3.655	0.115	

Readability values in all seven indices (Flesch-Kincaid level, Gunningfox index, Coleman-Liau index, SMOG index, Automated readability index, FORCAST grade level, Flesch reading ease) were significantly different when comparing Readable.com to ChatGPT (p<0.05). ChatGPT consistently determined readability to be at a lower reading level than the values determined by Readable.com (Table 2). The findings suggest a lack of concurrence between ChatGPT's algorithm and that of reputable tools, such as Readable.com.

**Table 2:** The mean readability of texts analyzed by Readable.com and ChatGPT was compared using Student's t-test, and it was found that all the means were significantly different.

Index	Reviewer	N	Mean	Std. Error Mean	p-value
Flesch-Kincaid Level	Human	9	10.8011	0.41134	0.002
	ChatGPT	9	8.9111	0.30887	
Gunning Fox Index	Human	9	13.6656	0.4042	0.001
	ChatGPT	9	11.2389	0.2914	
Coleman-Liau Index	Human	9	12.7367	0.29822	0.001
	ChatGPT	9	9.9056	0.31639	
SMOG Index	Human	9	13.3322	0.3746	0.001
	ChatGPT	9	9.4556	0.3338	
Automated Readability Index	Human	9	11.0811	0.45869	0.007
	ChatGPT	9	9.4667	0.24944	
FORCAST Grade Level	Human	9	11.3356	0.13822	0.001
	ChatGPT	9	8.6722	0.1706	
Flesch Reading Ease	Human	9	46.6433	1.83603	0.001
	ChatGPT	9	64.5167	0.93775	

In our previous study, we determined a significant difference in DISCERN scores between articles from private clinic versus academic or news websites (p<0.001) [3], suggesting that online information from private clinics was more biased and misleading. The analyzed sample comprised nine articles, of which four originated from private clinics and medical practices and five from academic institutions or news outlets. In this study, ChatGPT's DISCERN scores did not differ between private and academic/news sources (p=0.167), suggesting that ChatGPT was unable to accurately identify poor-quality information sources regarding shock wave therapy as a treatment for erectile dysfunction (Table 3).

**Table 3:** Using a one-way ANOVA and Tukey post-hoc analysis, we found no significant difference in ChatGPT-calculated DISCERN scores between academic/news and private clinic sources. * The mean difference is significant at the 0.05 level.

Reviewer and Article Type	Reviewer and Article Type	Mean Difference	Std. Error	p-value	95% Confidence Interval	
					Lower Bound	Upper Bound
Human Academic	Human Private	1.17000*	0.21542	<0.001	0.5975	1.7425
	ChatGPT Academic	0.12118	0.22707	0.95	-0.4823	0.7246
	ChatGPT Private	.57356*	0.21542	0.049	0.0011	1.146
Human Private	Human Academic	-1.17000*	0.21542	<0.001	-1.7425	-0.5975
	ChatGPT Academic	-1.04882*	0.21542	<0.001	-1.6213	-0.4763
	ChatGPT Private	-.59644*	0.2031	0.025	-1.1362	-0.0567
ChatGPT Academic	Human Academic	-0.12118	0.22707	0.95	-0.7246	0.4823
	Human Private	1.04882*	0.21542	<0.001	0.4763	1.6213
	ChatGPT Private	0.45238	0.21542	0.167	-0.1201	1.0249
ChatGPT Private	Human Academic	-.57356*	0.21542	0.049	-1.146	-0.0011
	Human Private	.59644*	0.2031	0.025	0.0567	1.1362
	ChatGPT Academic	-0.45237	0.21542	0.167	-1.0249	0.1201

## Discussion

**Figure 1:**
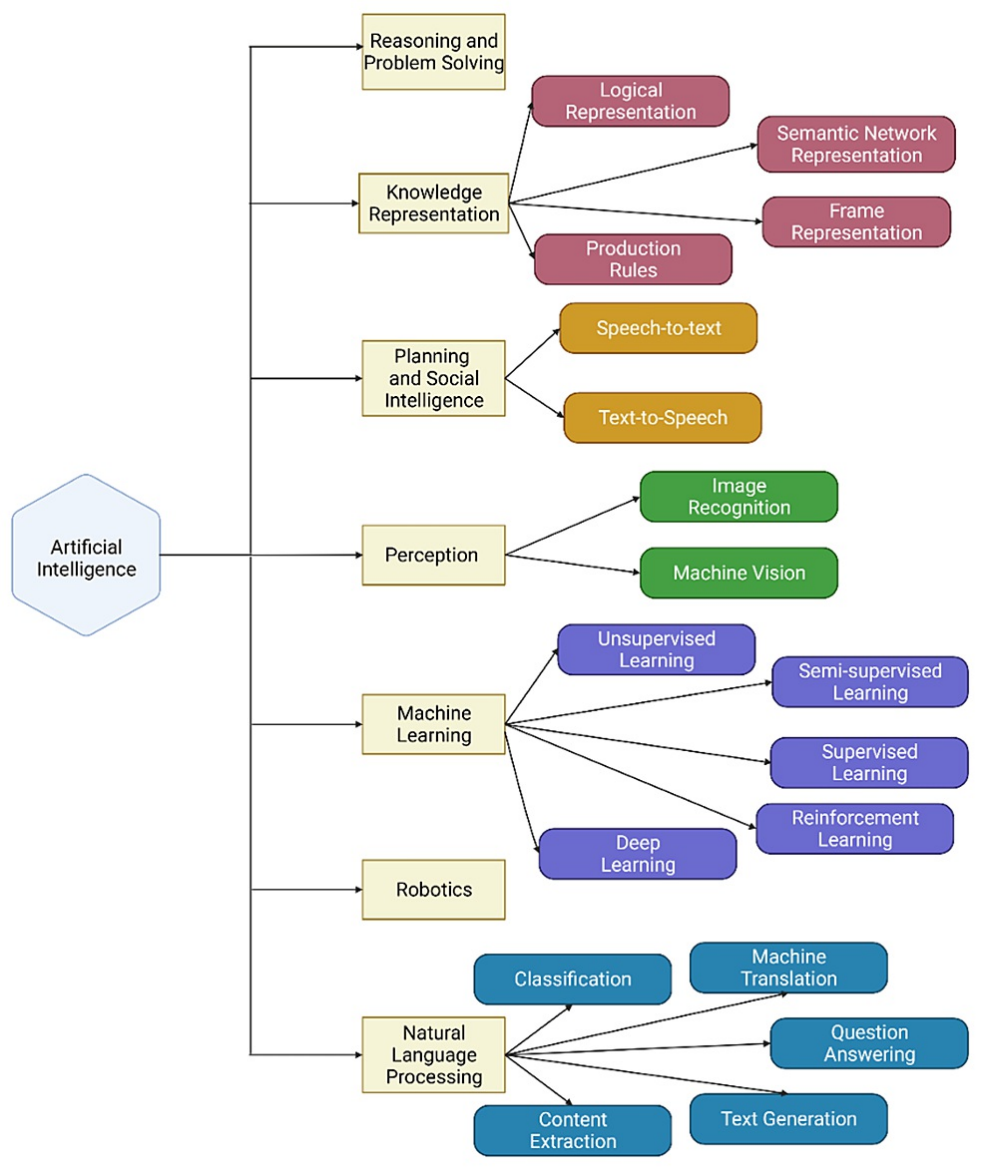
Subclasses of artificial intelligence. Conversational generative tre-trained transformer (ChatGPT) falls under deep learning.

The incorporation of AI (Figure 1) into the healthcare industry is seemingly inevitable. AI may play a role in various healthcare settings, including clinical practice, research investigation, patient education, peer review [14], and surgical technology [13-15]. Specifically, ChatGPT has become a powerful tool both for patients and physicians in both acquiring and distributing information. It is our responsibility as academic urologists to investigate the role of emerging AI technology to determine how we can best enhance patient care and research endeavors. Given the growing volume of medical content online and on social media platforms, there is a growing need for efficient and efficacious ways of evaluating the quality and readability of such medical content to ensure patients are receiving appropriate information.

To our knowledge, this study represents the first to specifically compare the performance of ChatGPT to human reviewers in evaluating the quality of online medical content, particularly that related to men’s health. Continued research is warranted to understand the implications of ChatGPT’s role in evaluating online medical content. Perhaps, if proven to be efficacious and accurate, it may lead to the ultimate development of AI plugins such as the DISCERN tool geared toward enhancing patient understanding of complex medical information across social media platforms and web browsers.

Restorative therapies for erectile dysfunction have attracted significant interest and research attention within men’s health with multiple ongoing clinical trials and many unanswered questions [16,17]. A high prevalence of poor-quality information online poses a particular challenge to today’s urologists [16,17]. We theorized that AI may be able to efficiently assess and discern credible information among online text sources with similar efficacy as human reviewers. Our study findings indicate that ChatGPT may not yet be able to complete this task, as well as humans. Specifically, ChatGPT-generated inconsistent DISCERN scores, upon repeat queries, overstated information quality and underscored information readability levels compared with human reviewers. Nonetheless, all queries performed with ChatGPT were successful, quick, and efficient, suggesting that there is a strong potential for AI to improve with continued exposure to content. Perhaps AI can achieve or surpass the ability of human reviewers to evaluate online medical content.

It remains unclear why there exists a discrepancy between Readable.com versus AI rating of readability. The effectiveness of classifying a text into readability levels depends on various factors, including the selection of the dataset, the choice of algorithm, and the selection of features to be extracted from the text [18]. AI algorithms may possess unique qualities that distinguish them from conventional readability tools that have been in use for a significant period.

This study is not without limitations. Given the unverified and automated nature of the ChatGPT platform, it may be difficult to draw conclusions regarding readability. Furthermore, given that this was a cross-sectional study, we understand that the search results yielded are based on a specific point in time and do not reflect the identical search circumstances of each patient. However, despite these limitations, the present data shed light on the potential role and feasibility of AI’s ability to evaluate the quality and readability of online medical content.

## Conclusions

ChatGPT is a promising AI tool that can be utilized to evaluate the quality and readability of online medical text. However, in its current state, it does so with less efficacy when compared to that of human reviewers and readability assessment tools, such as Readable.com.

ChatGPT's evaluation of current articles from websites regarding shockwave therapy, as a treatment for erectile dysfunction, generates overstated quality metrics and readability scores at a lower reading level than that of other online readability assessment tools. Thus, more investigation is warranted to both optimize ChatGPT’s medical content evaluation capabilities and to elucidate its role in enhancing patient access to high-quality information. Incorporating DISCERN tools and other established reading assessment tools into future ChatGPT software may be necessary to enhance its ability to evaluate the quality and readability of textual content and thus improve the efficacy of AI in this domain.

## References

[1] Rowlands G, Shaw A, Jaswal S, Smith S, Harpham T (2017). Health literacy and the social determinants of health: a qualitative model from adult learners. Health Promot Int.

[2] Nutbeam D, Lloyd JE (2021). Understanding and responding to health literacy as a social determinant of health. Annu Rev Public Health.

[3] Reddy RV, Golan R, Loloi J, Diaz P, Saltzman RG, Watane A, Ramasamy R (2022). Assessing the quality and readability of online content on shock wave therapy for erectile dysfunction. Andrologia.

[4] Siegal AR, Ferrer FA, Baldisserotto E, Malhotra NR (2023). The assessment of TikTok as a source of quality health information on varicoceles. Urology.

[5] Charnock D, Shepperd S, Needham G, Gann R (1999). DISCERN: an instrument for judging the quality of written consumer health information on treatment choices. J Epidemiol Community Health.

[6] Kaicker J, Borg Debono V, Dang W, Buckley N, Thabane L (2010). Assessment of the quality and variability of health information on chronic pain websites using the DISCERN instrument. BMC Med.

[7] Khan RA, Jawaid M, Khan AR, Sajjad M (2023). ChatGPT - reshaping medical education and clinical management. Pak J Med Sci.

[8] Johnson SB, King AJ, Warner EL, Aneja S, Kann BH, Bylund CL (2023). Using ChatGPT to evaluate cancer myths and misconceptions: artificial intelligence and cancer information. JNCI Cancer Spectr.

[9] Juhi A, Pipil N, Santra S, Mondal S, Behera JK, Mondal H (2023). The capability of ChatGPT in predicting and explaining common drug-drug interactions. Cureus.

[10] Sallam M (2023). ChatGPT utility in healthcare education, research, and practice: systematic review on the promising perspectives and valid concerns. Healthcare (Basel).

[11] Biswas SS (2023). Role of Chat GPT in public health. Ann Biomed Eng.

[12] Savage N (2023). Drug discovery companies are customizing ChatGPT: here's how. Nat Biotechnol.

[13] Golan R, Reddy R, Muthigi A, Ramasamy R (2023). Artificial intelligence in academic writing: a paradigm-shifting technological advance. Nat Rev Urol.

[14] Golan R, Reddy R, Deebel NA, Ramasamy R, Harris AM (2023). Peer review: a process primed for quality improvement?. J Urol.

[15] Stone L (2021). The dawning of the age of artificial intelligence in urology. Nat Rev Urol.

[16] Saltzman RG, Golan R, Masterson TA 3rd, Sathe A, Ramasamy R (2022). Restorative therapy clinical trials for erectile dysfunction: a scoping review of endpoint measures. Int J Impot Res.

[17] Khodamoradi K, Dullea A, Golan R, Molina M, Arora H, Masterson TA, Ramasamy R (2022). Platelet rich plasma (PRP) growth factor concentration varies in men with erectile dysfunction. J Sex Med.

[18] Alotaibi S, Alyahya M, Al-Khalifa H, Alageel S, Abanmy N (2016). Readability of Arabic medicine information leaflets: a machine learning approach. Procedia Computer Science.

